# Vitrification for cryopreservation of 2D and 3D stem cells culture using high concentration of cryoprotective agents

**DOI:** 10.1186/s12896-020-00636-9

**Published:** 2020-08-26

**Authors:** Young-Hoon Jeong, Ukjin Kim, Seul-Gi Lee, Bokyeong Ryu, Jin Kim, Artyuhov Igor, Jong Soo Kim, Cho-Rok Jung, Jae-Hak Park, C-Yoon Kim

**Affiliations:** 1 Department of Stem Cell Biology, School of Medicine, Konkuk University, Seoul, 05029 Republic of Korea; 2 Department of Laboratory Animal Medicine, Research Institute for Veterinary Science, BK21 PLUS Program for Creative Veterinary Science Research, College of Veterinary Medicine, Seoul National University, Seoul, 08826 Republic of Korea; 3 Kriorus, Klimentovsky Per, 115184 Moscow, Russia; 4 Gene Therapy Research Unit, Korea Research Institute of Bioscience and Biotechnology, Daejeon, 34141 Republic of Korea

**Keywords:** Vitrification, Cryoprotective agent, In vitro, MSC, Spheroid

## Abstract

**Background:**

Vitrification is the most promising technology for successful cryopreservation of living organisms without ice crystal formation. However, high concentrations (up to ~ 6–8 M) of cryoprotective agents (CPAs) used in stem cell induce osmotic and metabolic injuries. Moreover, the application of conventional slow-freezing methods to cultures of 3-D organoids of stem cells in various studies, is limited by their size.

**Results:**

In this study, we evaluated the effect of high concentrations of CPAs including cytotoxicity and characterized human mesenchymal stem cell (MSC) at single cell level. The cell viability, cellular damage, and apoptotic mechanisms as well as the proliferation capacity and multipotency of cells subjected to vitrification were similar to those in the slow-freezing group. Furthermore, we identified the possibility of vitrification of size-controlled 3-D spheroids for cryopreservation of organoid with high survivability.

**Conclusions:**

Our results demonstrate successful vitrification of both single cell and spheroid using high concentration of CPAs in vitro without cytotoxicity.

## Background

Organ cryopreservation is one of the most promising methods of storage and long-term preservation of transplantable cells and tissues. Cells, tissues, and organs for transplantation at cryogenic temperature (i.e., liquid nitrogen at − 196 °C) can be stored indefinitely, theoretically [1]. Cryogenic technology has the potential to revolutionize the overall status of organ transplantation in the medical field. Recently, organoids mimicking human organs have been widely studied to recapitulate the process of organogenesis in vitro. However, cryoprotective agents (CPAs) are hard to permeate organoids due to their strong cell-to-cell junctions and 3D structures without blood vessels compared with tissues or organs [2]. Moreover, ice crystal formation can cause severe cellular damage and destroy complex macroscopic tissues in preserved organs, and therefore, it is difficult to cryo-preserve an intact organoid.

Vitrification is a novel approach to cryopreservation that facilitates freezing of living cells without crystallization. Vitrification simplifies and enhances cryopreservation by eliminating mechanical injury induced by ice crystal formation, and provides the optimal range of ‘critical cooling & rewarming rate’ for various cells and tissues [3]. However, the high concentration of CPAs (up to ~ 8 M) induces osmotic effects during freezing and rewarming. High concentration of CPAs increases the risk of toxicity to cells and tissues compared with conventional slow freezing via non-vitrification, using low concentrations of CPAs [4, 5]. Such toxicity issues need to be addressed in cryopreservation studies [6].

Several studies have reported crystallization and thermomechanical characteristics of high-concentration CPAs including dimethyl sulfoxide (DMSO), ethylene glycol (EG) and propylene glycol (PG) currently used in tissue vitrification [7]. The general cytotoxicity of single permeable CPAs such as DMSO and EG has also been reported in various studies [8]. However, the in vitro toxicity of high concentration CPA solutions at the single-cell level or spheroids has been rarely reported [9, 10]. In this study, we evaluated the cytotoxicity of CPAs at a single-cell level and utilized vitrification methods for single-cell cryopreservation using in vitro cultures. Furthermore, we fabricated spheroids of various sizes using Human adipose-derived mesenchymal stem cells (MSC) loaded with a high concentration of CPAs followed by vitrification to form a glassy phase. After the rewarming step, the viability of spheroids in the vitrification group was higher than in the non-vitrified group as well as no significant differences were detected in the vitrified group compared with the vitrified group demonstrating lack of cytotoxicity. As the size of spheroids increased, dead cells in the non-vitrified group increased in core size. Our results suggest that vitrification using a high concentration of CPAs facilitated successful cryopreservation of cell aggregates more efficiently than slow freezing.

## Results

### Morphology and viability of vitrified-warmed cells

**Fig. 1:**
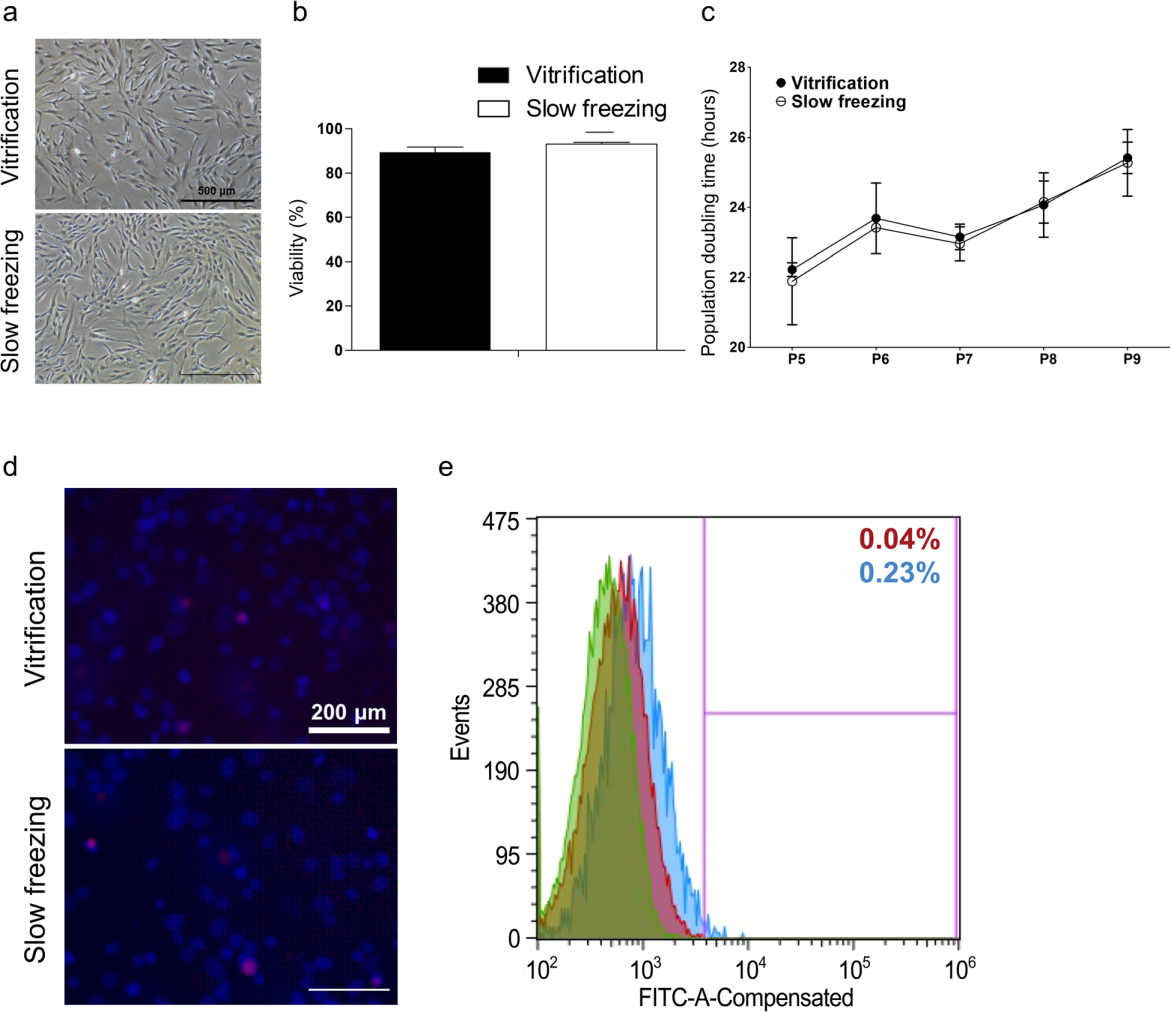
Cellular characteristics after re-warming compared with vitrification and slow-freezing method. **a** Morphology and **b** viability of MSCs after warming either vitrified or non-vitrified groups using trypan blue staining (left and middle) and **c** population doubling time (right). **d** The DNA fragmentation of each cell line by TUNEL assay (blue: cell, red: DNA strand breaks) and **e** the measurement of intracellular reactive oxygen species levels (green: unfrozen control, red: vitrification, blue: slow freezing)

To investigate morphology and viability of vitrified-warmed cells, we had frozen each cell line by using vitrification or non-vitrified method. In normal culture, MSC showed heterogenous fibroblast-like, elongated, and spindle-shaped single nuclear features following subsequent culture. Cells after warming in the vitrification or non-vitrified group showed fibroblast-like morphology and growth patterns similar to non-cryopreserved cells (Fig. 1a). Viabilities of vitrified MSC (v-MSC) and non-vitrified MSC (n-MSC) after warming were 89.4 ± 4.2% and 93.2 ± 1.2%, respectively (Fig. 1b). Although the viability of vitrified MSC was slightly decreased compared to that of the non-vitrified group, no significant difference was observed between two groups. Furthermore, population doubling time of warmed cells also observed no significant difference until 5 passages (Fig. 1c). These results revealed that vitrification could be as efficient as slow freezing in terms of maintaining cell viability for normal cell culture after warming.

### Detection of apoptotic DNA fragmentation (TUNEL assay)

To compare DNA fragmentation of each cell line between vitrified and non-vitrified groups, we performed TUNEL assay using fluorescence microscope. Results showed that there was no significant difference in the number of TUNEL^+^ cells between vitrified and non-vitrified groups (Fig. 1d). These data demonstrate that vitrification does not impair cellular DNA. Thus, it might be a safer approach of cell preservation than non-vitrified method due to the intracellular crystallization by slow freezing.

### Intracellular levels of reactive oxygen species after Vitrification

We investigated cellular ROS to identify osmotic stress or CPA toxicity caused oxidative stress. There was no significant difference in ROS level among vitrified and non-vitrified groups at all cell lines (Fig. 1e). These results demonstrate that ROS is not generated by vitrification when cells were frozen and warmed rapidly.

### Characterization of human adipose-derived Mesenchymal stem cells

**Fig. 2:**
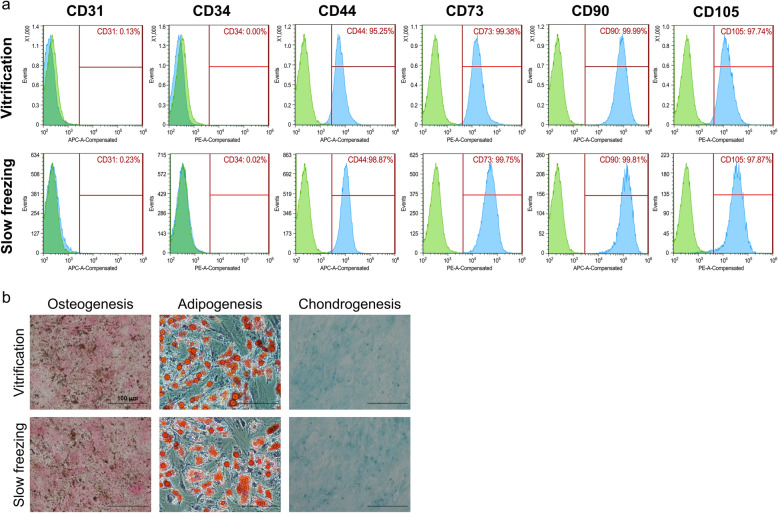
Characterization of AD-MSCs after rewarming. **a** Negative markers for CD31 and CD34, positive markers for CD44, CD73, CD90, and CD 105. **b** Staining for multilineage differentiation of AD-MSCs

Cell surface antigen profiles of v-MSC and n-MSC were characterized by flow cytometry. All groups displayed a general MSC antigen profile that exhibited CD44, CD73, CD90, and CD105 expression, while CD31 and CD34 for the negative markers were not detected (Fig. 2a). Both groups expressed all proper markers of MSCs without expressing any markers of differentiation. In order to further investigate the differentiation potential of MSCs, cells were induced to differentiate into adipocytes, chondrocyte, and osteoblasts based on oil red O staining, Alcian blue staining, Von kossa staining, respectively (Fig. 2b). These indicated that there was no significant difference between v-MSCs and n-MSCs in terms of differentiation potential or their characteristics.

### Viability of spheroids by size after rewarming

**Fig. 3:**
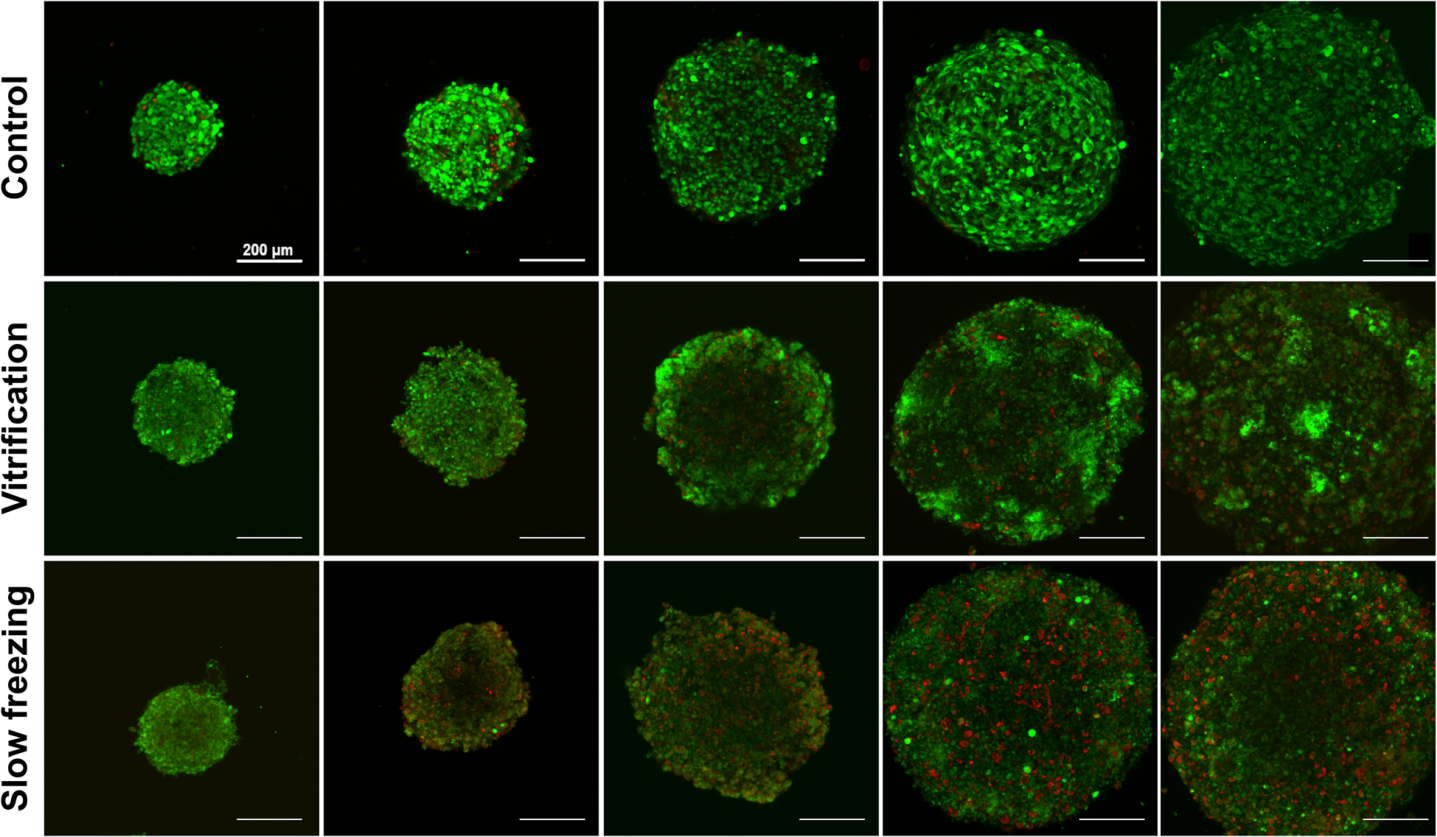
Viability of various size of spheroids after rewarming via live-dead staining (green: live cells, red: dead cells). From left, the size of each spheroid (200, 300, 500, 700, 900 μm) and the number of cells (10,000, 30,000, 50,000, 70,000, 100,000 cells/spheroid)

Next, we fabricated spheroids of various sizes (200–900 μm) and confirmed the viability of vitrification and non-vitrification groups after the rewarming procedure. After freezing and rewarming steps, we visualized the cell survival using live-dead staining procedures. As shown in Fig. 3, in the control group, most of the cells were living (green fluorescence), and few of the dead cells (red fluorescence) were observed. On the other hand, unlike the normal 2D cell culture results, the non-vitrified (slow freezing) group showed excessive cell death in the core region; and the vitrified group showed relatively mild cell death. These findings demonstrate that the viability of cell aggregates was enhanced in the vitrification group after rewarming because high concentrations of CPAs may permeate to spheroids core and protect cell death.

### Quantitative real-time polymerase chain reaction

**Fig. 4:**
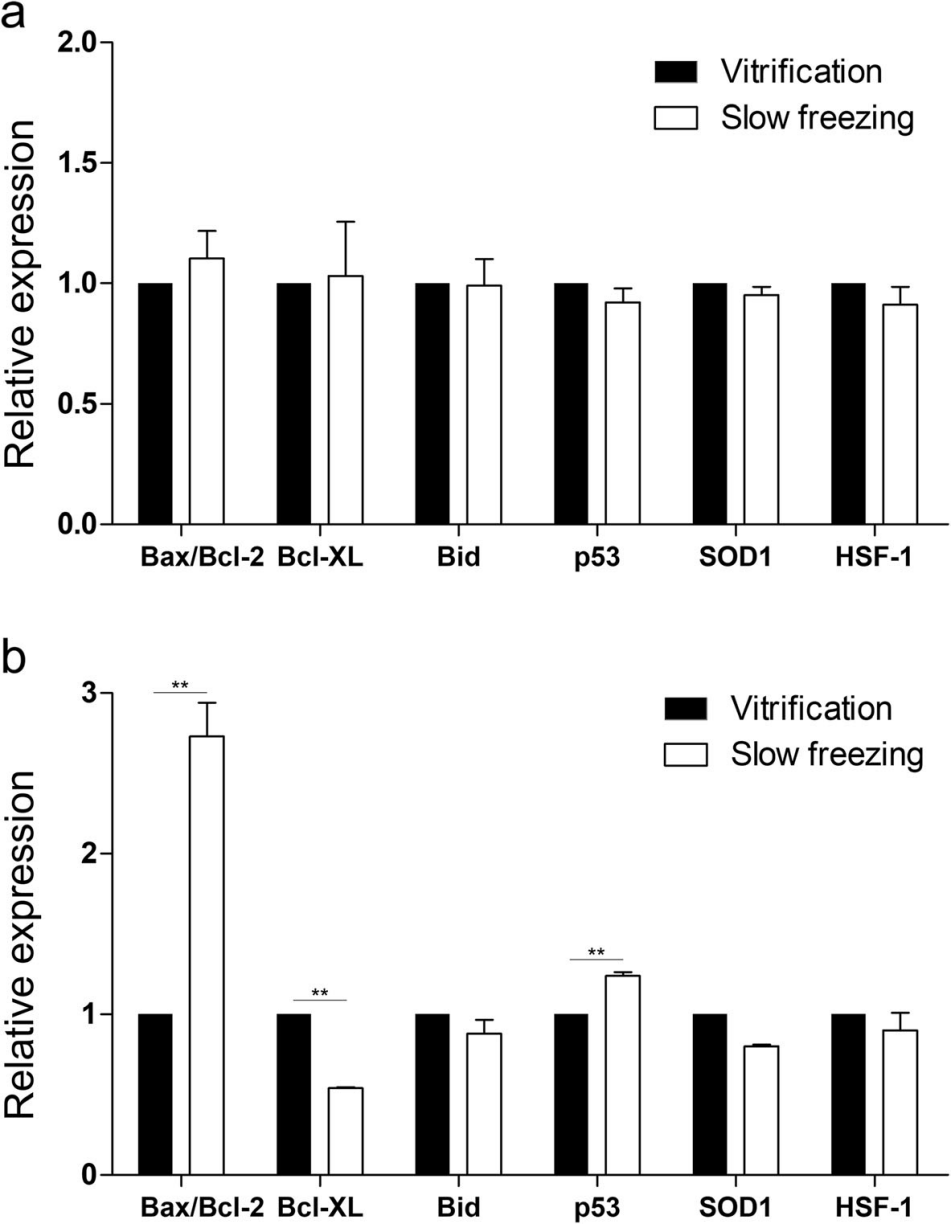
Quantitative real-time polymerase chain reaction (RT-PCR) analysis for apoptosis, oxidative stress, and heat shock damage after rewarming at single cell level (**a**) and spheroids (**b**). (*n* ≥ 3). **p < 0.01 relative to the vitrified group

We investigated RNA expression levels to analyze whether vitrification affected cell viability. Unfortunately, there were no significant differences between vitrified and non-vitrified groups at single cell levels (Fig. 4a). We also analyzed various gene expression of each group of spheroids to confirm the increased viability after vitrification of spheroids. Bax/Bcl-2 ratios non-vitrified spheroids showed higher Bax/Bcl-2 levels compared with vitrified spheroids (Fig. 4b). Bcl-xL, a member of the Bcl-2 family of proteins was considerably upregulated at vitrified spheroids and Bid, a pro-apoptotic Bcl-2 protein containing only the BH3 domain were not significant difference between vitrified and non-vitrified spheroids. Moreover, p53 related to apoptosis was significantly increased in non-vitrified spheroids, demonstrating that the apoptosis by cryo-damage intensified when cells were frozen slowly at cell aggregates. We further analyzed SOD1 gene to confirm generation of oxidative stress caused by removing ROS. Results showed that both non-vitrified and vitrified spheroids had similar expression levels. Likewise, HSF-1 had identical tendency to SOD1 expression. Taken together, toxicity of high-CPAs for vitrification does not affect cell death at single cell level, but rather apoptosis related genes were significantly upregulated when cells were aggregated and frozen slowly.

## Discussion

In this study, we established the role of vitrification at single-cell level and spheroids in vitro using high concentrations of CPAs. Several studies reported that high concentrations of CPAs such as DMSO, EG, and other compounds are used for organ storage; however, relatively low concentrations of CPAs are used for vitrification of single cells such as oocytes [11, 12]. Vitrification is performed using a mixture of permeable CPA components based on DMSO, EG, and/or PG at concentrations up to 8 M to avoid ice crystal formation. In addition, although many studies have reported the CPA composition and concentration [13], a specific approach for equilibration and replacement is required for in vitro single-cell applications. Therefore, the evaluation of cytotoxicity associated with CPAs is key to overcome the limitations of single-cell cryopreservation because of the high concentration of CPAs used for organ cryopreservation.

Differences in osmotic pressure are a serious hindrance to the application of high concentrations of CPAs in cells. Therefore, the control of osmotic pressure is one of the most important factors for successful preservation. Moreover, buffering the control of osmotic pressure by regulating the CPA concentration is essential to minimize the damage [14]. This step is not implemented during conventional slow freezing. It entails a gradual decrease in the density of solution using a high concentration of sucrose to alleviate the osmotic pressure induced by high concentrations of CPAs. If this process is not accurate or efficient, cells are damaged by differences in osmotic pressure during the warming stage.

The cell survival rates, cellular damage, and apoptosis-related gene expression suggest that the viability after rewarming was not significantly different between the two groups. After rewarming, the population doubling time of the vitrification group was similar to that of the non-vitrified group by passage 5. Moreover, the vitrification group showed a similar expression of genes involved in oxidative stress, heat shock damage, and apoptosis. In addition, no significant difference in the detection of DNA fragmentation by TUNEL assay and direct ROS measurement was observed. These results indicated that vitrification does not affect molecular damage at single cell levels even with high concentration of CPAs. The v-MSCs retained normal differentiation potential and their characteristic features (Fig. 2). Additionally, we investigated cell viability, cellular damage and gene expression using other cell lines, human dermal fibroblast (hDF) and 293FT cells to ensure safety of high-CPAs at single cell level. Viabilities of vitrified hDF (v-hDF) and non-vitrified hDF (n-hDF) were 92.8 ± 1.8% and 94.6 ± 1.5%, respectively. Viabilities of vitrified 293FT (v-293FT) and non-vitrified 293FT (n-293FT) were 95.8 ± 0.3% and 95.9 ± 1.6%, respectively (Supplementary Fig. 1A, B). Also, no significant difference in DNA fragmentation and intracellular ROS level was detected between vitrified and non-vitrified groups of hDF and 293FT cells (Supplementary Fig. 1C, D). These findings demonstrate that vitrification not only does not induce cellular damage or sustained proliferation capacity but also does not influence cell characteristics or differentiation potential.

As the organoid system is refined and increasingly utilized, many trials have investigated the therapeutic applications of organoids [15, 16]. However, large-sized organoids compared with single cells cannot be easily preserved intact for long periods of time, and long-term cultures are expensive. In this study, we cryopreserved size-controlled spheroids and identified their viability after rewarming procedures. As shown in Fig. 3, the viability of size-controlled spheroids was increased when spheroids were frozen via vitrification compared with slow freezing and, as the size of spheroids increases, the viability seems to be increased. Moreover, we confirmed that in spheroids prepared using other cells, hepatocellular carcinoma cell line HepaRG, a large amount of cell death was found in the core region (Supplementary Fig. 2A) as well as the expression of apoptosis associated genes was upregulated (Supplementary Fig. 2B). We speculate that high concentrations of CPAs permeate spheroids to the core region homogeneously, which explains the enhanced viability of the vitrified groups after rewarming compared with the poor penetration of spheroids via slow freezing.

## Conclusions

In conclusion, our results demonstrate that vitrification does not induce additional damage in the single cell. The vitrification strategy facilitated successful cryopreservation of 3D spheroids with increased survivability when compared with the conventional slow-freezing technique. It may represent a promising technology for advanced organ vitrification studies.

## Methods

### Cell Source & culture

MSC purchased from Lonza (PT-5006; Walkersville, MD) were seeded at the density of 4500 cell/cm^2^ on polystyrene tissue-culture dishes and cultured in Dulbecco’s Modified Eagle’s Medium (DMEM; Thermo Fisher Scientific, Waltham, MA, USA) including GlutaMAX supplemented with 10% fetal bovine serum (FBS; Thermo Fisher Scientific) and 1% of penicillin-streptomycin (P/S; Thermo Fisher Scientific). Cells were maintained at 37 °Cin a humidified atmosphere containing 5% CO_2_. The culture medium was changed every 2 days. Cells were continuously cultured in the culture medium described above until reaching 90% confluency. Cells for all experiments were used at passage 5.

### Generation of MSC spheroids

To generate spheroids, MSCs at passage 5 were plated in suspension using hanging drop method in 25 μL of culture medium containing 10,000–100,000 cells/drop for up to 7 d. Cells were incubated in MSC culture medium, and placed in a humidified atmosphere at 37 °C and 5% CO_2_. MSC spheroids were identified using an inverted microscope (Nikon, Chiyoda-ku, Japan).

### Vitrification of cells

Vitrification of cells was performed using a two-step exposure vitrification solution, equilibration and vitrification solutions [17]. The equilibration solution was 1.4 M DMSO (Sigma-Aldrich, St. Louis, MO, USA) and EG (Sigma-Aldrich) based on Dulbecco’s phosphate buffered saline (DPBS; Thermo Fisher Scientific) containing 20% (v/v) FBS. The vitrification solution was comprised of 2.8 M DMSO, 2.7 M EG, 2.8 M formamide (F; Sigma-Aldrich), and 70 g/L PVP K12 (Thermo Fisher Scientific) based on LM5 carrier solution [4]. In addition, we slightly modified LM5 carrier solution that contained 0.3 M sucrose instead of glucose, mannitol, and lactose. First, each pellet of cells about 500,000 cells per vial detached by 0.25% trypsin-EDTA (Thermo Fisher Scientific) were suspended in equilibration solution for 10 min and then blended with vitrification solution within 1 min (Table 1). Suspended cells were immediately transferred to 1.5 ml cryovials (Nunc, Rochester, MN, USA) and plunged directly into liquid nitrogen. Likewise, vitrification of MSC spheroids was performed using a two-step exposure vitrification solution as described above. Every spheroid was cryopreserved about 10 spheroids per vial. Two weeks later, cells were immediately warmed by immersing vials in a water bath at 37 °C. Warmed cells were suspended serially in 0.5, 0.25, and 0 M sucrose in DPBS containing 20% FBS for 5 min, respectively (Table 1). Finally, each cell line was resuspended and cultured with culture medium.

**Table 1:** Stepwise CPA introduction and withdrawal

CPA addition steps		CPA washout steps		
Equilibration solution 10 min	CPA (VM3) 1 min	0.5 M sucrose 5 min	0.25 M sucrose 5 min	0 M sucrose 5 min

### Slow freezing of cells

To compare the survival rate after warming with vitrified cells, generally performed cryopreservation method was used as the control for non-vitrified cells. Briefly, pellets detached by 0.25% trypsin-EDTA were transferred to 1.5 ml cryovials containing 10% DMSO in FBS. Cryovials were sealed and frozen to − 80 °C in Mr. Frosty controlled cooling rate devices (Thermo Fisher Scientific). After 24 h, cryovials were transferred to liquid nitrogen. Two weeks later, cryovials were placed in a 37 °C water bath for warming and agitated until only a pea-sized piece of ice remained. Then, cells were centrifugated at 1000 rpm 25 °C, and resuspended and cultured with culture medium.

### Evaluation of cell viability and morphology

After warming, the survival rate was determined after trypan blue (Thermo Fisher Scientific) staining when cells were sequentially warmed as described above. Remaining cells were plated at a density of 4500 cells/cm^2^. Each cell line was observed with an inverted microscope (Nikon) after 1 day to confirm cell attachment to the culture dish. Spheroid viability was determined using LIVE-DEAD cell viability kit (Thermo Fisher Scientific). For live-dead assay, 15 min after adding 2 μM of calcein AM (green, live cells) and 4 μM of ethidium homodimer-1 (red, dead cells), spheroids were imaged using Zeiss 710 Confocal microscope (Carl Zeiss, Germany).

### Cell proliferation assay

MSCs were counted using a hemocytometer at the beginning and end of each passage. Population doubling time (PDT) was measured by the formulas PDT = ln2*T/ln (NT/N0), where NT is cell number at the end of a passage, N0 is the cell number at the seeding density, and T is culture time.

### TUNEL assay

DNA fragmentation was detected using terminal deoxynucleotidyl transferase 2-deoxyuridine 5-triphosphate (dUTP) nick end labeling (TUNEL; Roche, Indianapolis, IN, USA). Vitrified cells (v-cells) and non-vitrified cells (n-cells) were warmed as described above, fixed in 4.0% paraformaldehyde and subjected to TUNEL assay using a Click-iT™ Plus TUNEL Assay (Thermo Fisher Scientific) according to the manufacturer’s instructions. Images of TUNEL+ cells were acquired using a fluorescence microscope (Nikon).

### Measurement of intracellular reactive oxygen species

Reactive oxygen species (ROS) species were measured using a DCFDA cellular ROS detection assay kit (Abcam, Cambridge, MA, USA) following the manufacturer’s instructions. Briefly, after warming v-cells and n-cells, each pellet was then incubated in 1× Buffer containing 25 μM DCFDA at 37 °C for 45 min. ROS was measured by flow cytometry without wash steps and 30,000 labelled cells were acquired and analyzed using Becton Dickinson FACS Calibur.

### Quantitative real-time polymerase chain reaction

Total RNA of each sample was extracted using TRIzol Reagent (Invitrogen, Carlsbad, CA, USA) according to the manufacturer’s instructions. Then 1 μg of total RNA was transcribed into complementary deoxyribonucleic acid (cDNA) using a High-Capacity cDNA Reverse Transcription Kit (Thermo Fisher Scientific). Real-time PCR was performed using FastStart Essential DNA Green Master (Roche, Pleasanton, CA, USA) on a LightCycler 96 instrument (Roche). All PCR reactions were performed in triplicate. The mean cycle threshold (Ct) values of triplicate wells for each sample were collected and the expression data was normalized to the endogenous control glyceraldehyde 3-phosphate dehydrogenase (GAPDH). Target genes and associated primers are as follows: GAPDH sense 5′-GTCTGAACCATGAGAAGTATGA, GAPDH antisense 5′-CTTCCACGATACCAAAGTTGT, *Bax* sense 5′-GTCAGCTGCCACTCGGAAA, *Bax* antisense 5′-AGTAACATGGAGCTGCAGAGGAT, *Bcl-2* sense 5′-TCAGAGACAGCCAGGAGAAATCA, *Bcl-2* antisense 5′-CCTGTGGATGACTGAGTACCTGAA, *Bcl-XL* sense 5′-ATGGCAGCAGTAAAGCAAGC, *Bcl-XL* antisense 5′-CGGAAGAGTTCATTCACTACCTGT, *Bid* sense 5′-ACTGGTGTTTGGCTTCCTCC, *Bid* antisense 5′-ATTCTTCCCAAGCGGGAGTG, *SOD1* sense 5′-CTGAAGGCCTGCATGGATTC, *SOD1* antisense 5′-CCAAGTCTCCAACATGCCTCTC, *p53* sense 5′-CCCAAGCAATGGATGATTTGA, *p53* antisense 5′-GGCATTCTGGGAGCTTCATCT, *HSF1* sense 5′-GCCTTCCTGACCAAGCTGT, *HSF1* antisense 5′-AAGTACTTGGGCAGCACCTC.

### Characterization of MSC by flow cytometry

Cells were characterized as described previously [18]. Briefly, cells at ~ 90% confluence after passage were detached by 0.25% trypsin-EDTA. Pellets were resuspended in FACS buffer (DPBS solution including 0.5% bovine serum albumin (BSA) and 2 mM EDTA) and filtered using a premoistened 40-μm cell strainer. Cells were then labelled using each antibody of MSC surface markers according to the manufacturer’s instructions. The following antibodies were used: fluorochrome-conjugated antibodies for CD44-APC, CD73-PE, CD90-APC, CD105-PE (BD Biosciences, Bedford, MA, USA), and negative markers CD31 and CD34 conjugated to APC and PE (BD Biosciences). Corresponding IgG controls were prepared equally, and 30,000 labelled cells were acquired and analyzed using Becton Dickinson FACS Calibur.

### Evaluation of the differentiation potential of MSC

For the induction of osteoblasts, chondroblasts, and adipocytes, commercially available kits (Thermo Fisher Scientific) were used as described previously [18]. Briefly, cells under differentiation conditions were maintained in 12-well plates. Osteogenesis and chondrogenesis were induced for 21 days while adipogenic lineage was induced for 14 days. All experimental procedures were performed according to the manufacturer’s instructions. To evaluate each differentiation process, appropriate staining was performed. Oil Red O staining was used to detect intracellular lipid droplets. Von Kossa staining was performed to visualize extracellular mineralized matrix and Alcian blue staining was used to confirm the formation of proteoglycans. Images were analyzed using an inverted microscope (Nikon, Chiyoda-ku, Japan).

### Statistical analysis

All statistical analyses were performed using GraphPad Prism software version 5 (La Jolla, CA, USA). All statistical data are displayed as mean ± SEM. Statistical significance of experimental outcomes was determined using one-way ANOVA. Differences between experimental groups were considered significant when *p* < 0.05.

## Supplementary information

**Additional file 1: Supplementary Fig. 1.** Cellular characteristics after re-warming compared with vitrification and slow-freezing method using various cell lines. (A) Morphology and (B) viability of MSCs after warming either vitrified or non-vitrified groups using trypan blue staining. (C) The DNA fragmentation of each cell line by TUNEL assay (blue: cell, red: DNA strand breaks) and (D) the measurement of intracellular reactive oxygen species levels (green: unfrozen control). **Supplementary Fig. 2.** Survivability and various gene expression of rewarmed spheroids using hepaRG cell line. (A) Viability of the largest size of spheroids after rewarming via live-dead staining. (B) Quantitative real-time polymerase chain reaction (RT-PCR) analysis for apoptosis, oxidative stress and heat shock damage after rewarming. (*n* ≥ 3). *p < 0.05, relative to the vitrified group.

## Data Availability

Any information used and/or analyzed during this study is available from the corresponding author on reasonable request.

## Abbreviations

CPAs: Cryoprotective agents
MSC: Mesenchymal stem cells
DMSO: Dimethyl sulfoxide
EG: Ethylene glycol
PG: Propylene glycol
DMEM: Dulbecco’s modified Eagle’s medium
FBS: Fetal bovine serum
P/S: Penicillin-streptomycin
DPBS: Dulbecco’s phosphate buffered saline
F: Formamide
PDT: Population doubling time
dUTP: 2-deoxyuridine 5-triphosphate
TUNEL: Terminal deoxynucleotidyl transferase dUTP nick end labeling
v-cells: Vitrified cells
n-cells: Non-vitrified cells
ROS: Reactive oxygen species
cDNA: Complementary deoxyribonucleic acid
BSA: Bovine serum albumin
hDF: Human dermal fibroblast
v-hDF: Vitrified hDF
n-hDF: Non-vitrified hDF
v-293FT: Vitrified 293FT
n-293FT: Non-vitrified 293FT
