# Supplementary material for: Vitrification for cryopreservation of 2D and 3D stem cells culture using high concentration of cryoprotective agents
Source: BMC Biotechnol. 2020 Aug 26;20:45. doi: 10.1186/s12896-020-00636-9 (PMC7449025; doi:10.1186/s12896-020-00636-9)
Supplement: Supplementary file 1 — Additional file 1: Supplementary Fig. 1. Cellular characteristics after re-warming compared with vitrification and slow-freezing method using various cell lines. (A) Morphology and (B) viability of MSCs after warming either vitrified or non-vitrified groups using trypan blue staining. (C) The DNA fragmentation of each cell line by TUNEL assay (blue: cell, red: DNA strand breaks) and (D) the measurement of intracellular reactive oxygen species levels (green: unfrozen control). Supplementary Fig. 2. Survivability and various gene expression of rewarmed spheroids using hepaRG cell line. (A) Viability of the largest size of spheroids after rewarming via live-dead staining. (B) Quantitative real-time polymerase chain reaction (RT-PCR) analysis for apoptosis, oxidative stress and heat shock damage after rewarming. (n ≥ 3). *p < 0.05, relative to the vitrified group. [file 12896_2020_636_MOESM1_ESM.docx]

**
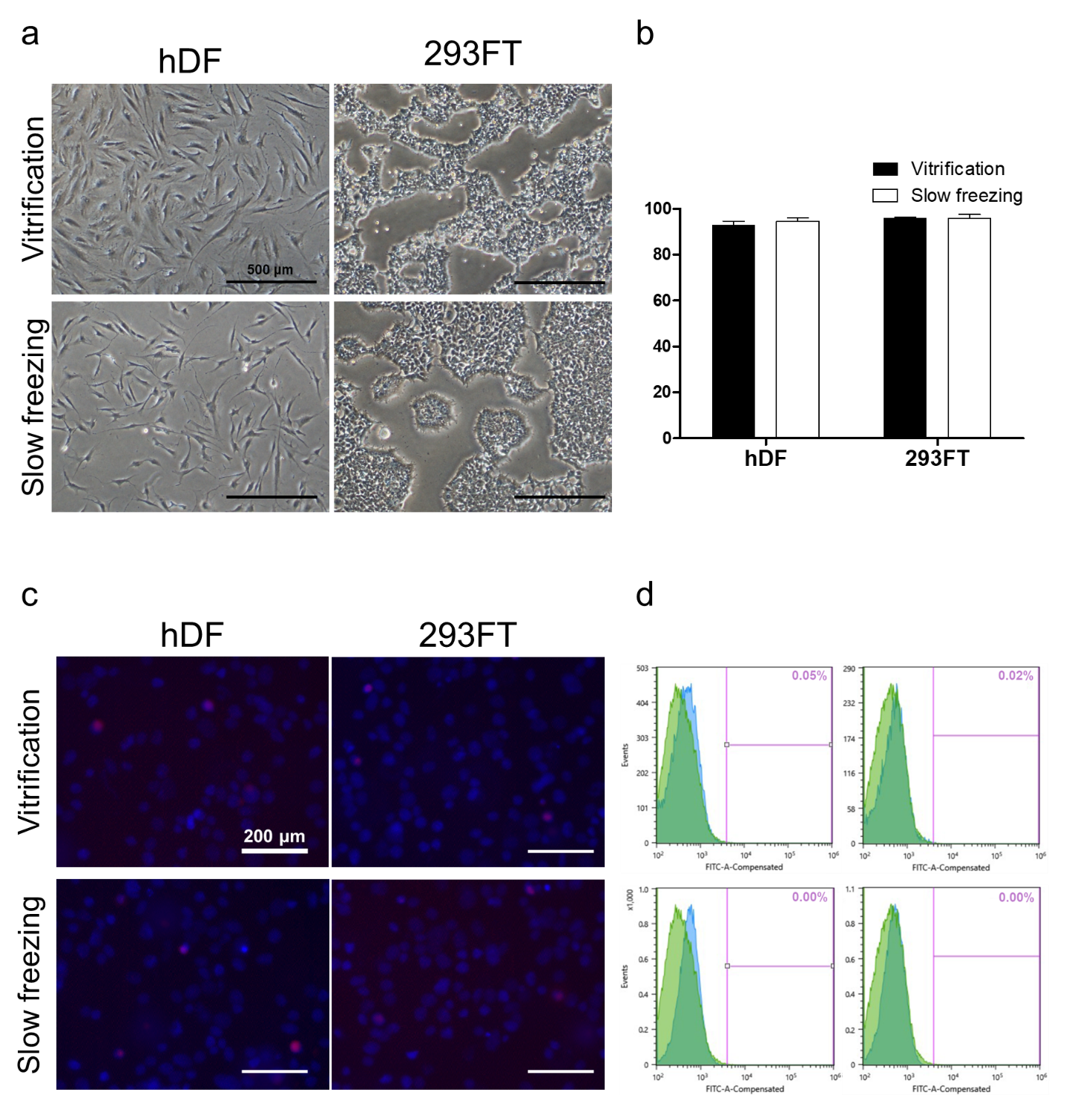
**

**Supplementary Fig. 1.** Cellular characteristics after re-warming compared with vitrification and slow-freezing method using various cell lines. (A) Morphology and (B) viability of MSCs after warming either vitrified or non-vitrified groups using trypan blue staining. (C) The DNA fragmentation of each cell line by TUNEL assay (blue: cell, red: DNA strand breaks) and (D) the measurement of intracellular reactive oxygen species levels (green: unfrozen control)

**
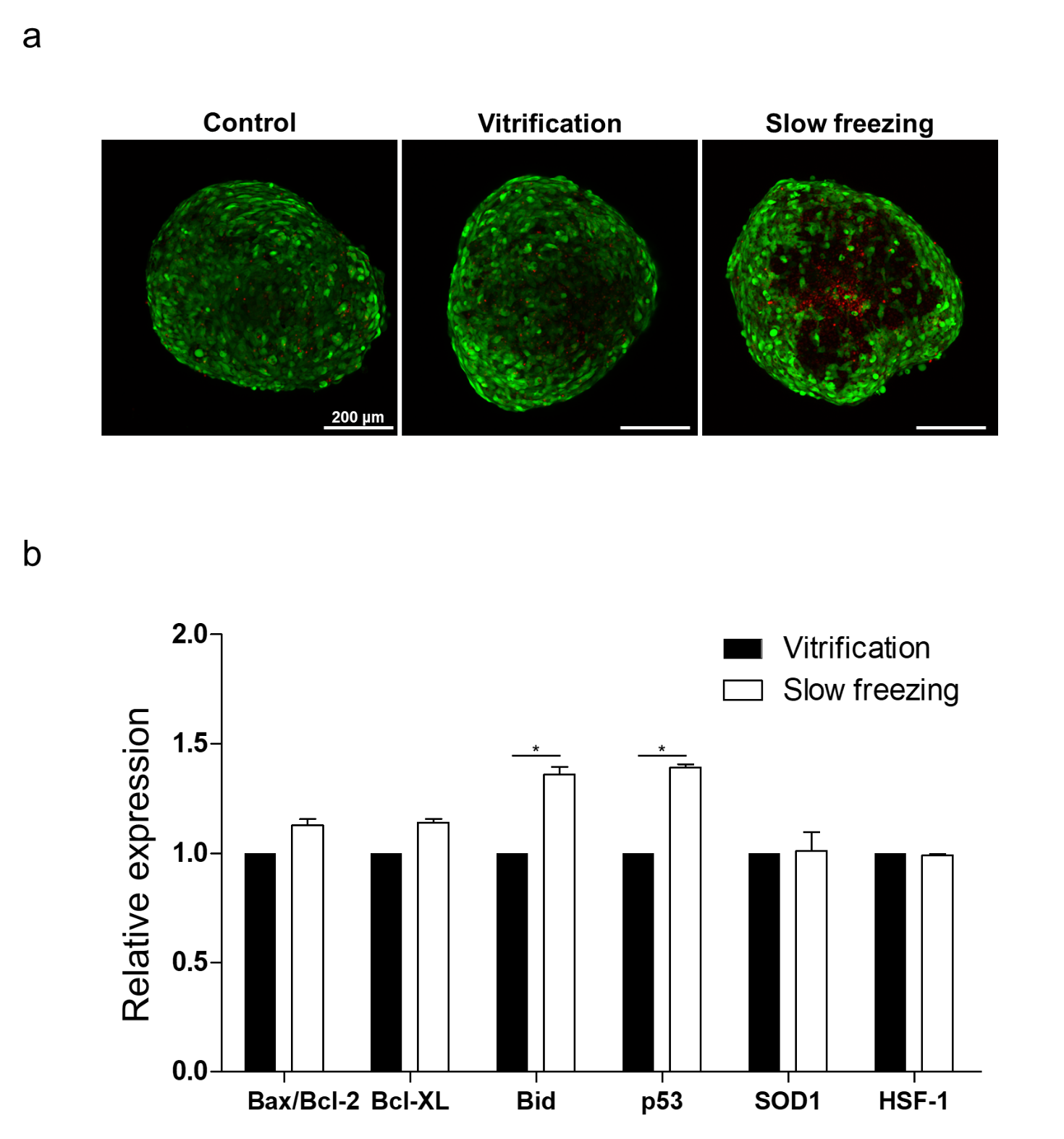
**

**Supplementary Fig. 2**. Survivability and various gene expression of rewarmed spheroids using hepaRG cell line. (A) Viability of the largest size of spheroids after rewarming via live-dead staining. (B) Quantitative real-time polymerase chain reaction (RT-PCR) analysis for apoptosis, oxidative stress and heat shock damage after rewarming. (n ≥ 3). *p < 0.05, relative to the vitrified group.
